# Complete chloroplast genome sequence of *Chrysosplenium ramosum* and *Chrysosplenium alternifolium* (Saxifragaceae)

**DOI:** 10.1080/23802359.2020.1790313

**Published:** 2020-07-15

**Authors:** Wen-jie Yan, Hong Liu, Tian-ge Yang, Rui Liao, Rui Qin

**Affiliations:** a College of health and environment, Beijing Union University, Beijing, China; b Hubei Provincial Key Laboratory for Protection and Application of Special Plant Germplasm in Wuling Area of China, Key Laboratory of State Ethnic Affairs Commission for Biological Technology, College of Life Science, South-Central University for Nationalities, Wuhan, China

**Keywords:** *Chrysosplenium*, chloroplast genome, phylogeny

## Abstract

The complete chloroplast genome of *Chrysosplenium ramosum* Maxim. and *Chrysosplenium alternifolium* L. were reported in this study. The chloroplast genomes were 153,460 bp for *C. ramosum* and 152,619 bp for *C. alternifolium*. LSC and SSC of 83,670 bp and 17,342 bp were separated by two IRs of 26,224 bp each in *C. ramosum*. While *C. alternifolium* contained IRs of 25,992 bp, LSC of 83,524 bp and SSC of 17,111 bp. The chloroplast genome of *C. ramosum* contains 112 unique genes, including 79 protein-coding genes, four ribosomal RNA genes, and 30 transfer RNA genes. And the chloroplast genome of *C. alternifolium* contains 112 unique genes, including 79 protein-coding genes, four ribosomal RNA genes, and 30 transfer RNA genes. In addition, the rps12 gene was recognized as a trans-spliced gene and 17 intron-containing genes were also detected.

*Chrysosplenium* L. (Saxifragaceae) comprises about 65–70 species in worldwide, mainly distributed in the northern hemisphere (Hara 1957; Soltis et al. 2001; Lan et al. 2018; Liao et al. 2019). Due to the different characters: four sepals of flowers, lack petals, and have two equal or distinctly unequal fruiting capsules, *Chrysosplenium* is important in the taxonomy of Saxifragaceae (Maximowicz 1872). *Chrysosplenium* is divided into two subgenera: *Alternifolia* Franchet and *Oppositifolia* Franchet according to leaf arrangement (Hara 1957). In this study, we report and characterize two chloroplasts genomes from two species: *Chrysosplenium ramosum* from subgenus *Oppositifolia* and *Chrysosplenium alternifolium* from subgenus *Alternifolia*. Using these data, we reconstruct the phylogenetic tree of this genus to reveal the relationship and provide useful information for further study of *Chrysosplenium*.

The materials of *C. ramosum* and *C. alternifolium* were collected from Jilin, China (E 127° 30′ 30″, N 42° 10′ 27″) and Shimane-ken, Japan (E 132° 36′ 11″, N 35° 08′ 22″). The voucher specimens were deposited at the Herbarium of South-Central University for Nationalities (HSN) and the specimen accession numbers are SJH2017052107372 and RG2019032810003. The complete genomic DNA of *C. ramosum and C. alternifolium* were extracted using CTAB method and sequenced using the Illumina platform at Biomarker Technologies (Beijing, China). After filtered the low-quality data and adaptors, the obtained clean data were aligned to *C. macrophyllum* (MK973001) with bwa-0.7.12. The aligned reads were then assembled with ABYSS-2.0.2 (Jackman et al. 2017) after the best Kmer was chosen with kmergenie (Medvedev and Chikhi 2014). Then, connected the overlap and scaffolding again by SSPACE_Standard_v3.0. Finally, the gaps were filled by Sanger.

The complete chloroplast genomes of these two species exhibited a general quadripartite structure. *C. ramosum* (MK973002) was 153,460 bp in length and included a large single-copy (LSC) region of 83,670 bp and a small single-copy (SSC) region of 17,342 bp separated by two reverse repeated regions of 26,224 bp, the average GC content was 37.38%. On the other hand, the complete chloroplast genome size of *C. alternifolium* (MT362050) was 152,619 bp in length, the average GC content was 37.47%. Separating LSC of 83,524 bp and SSC of 17,111 bp, a pair of IRs was 25,992 bp long in each. The chloroplast genome of *C. ramosum* contains 130 genes, including 85 protein-coding genes, eight ribosomal RNA genes, and 37 transfer RNA genes. The chloroplast genome of *C. alternifolium* contains 130 genes, including 85 protein-coding genes, eight ribosomal RNA genes, and 37 transfer RNA genes.

Phylogenetic analysis was performed using whole chloroplast coding sequences of *C. ramosum* and *C. alternifolium*, combining with three species of *Chrysosplenium*, 22 species of Saxifrageles, and two species of Buxaceae as outgroup. The phylogenetic relationships were reconstructed by means of maximum-likelihood (ML) with the model of GTR + F+R3 and Bayesian inference analysis using the Markov Chain Monte Carlo method with 200,000 generations and sampling trees every 100 generations. Based on the phylogenetic tree, we can see that the five species of *Chrysosplenium* were clustered to a clade (Figure 1). What’s more, the five species of *Chrysosplenium* seemed to divide into two clades by two subgenera.

**Figure 1.**
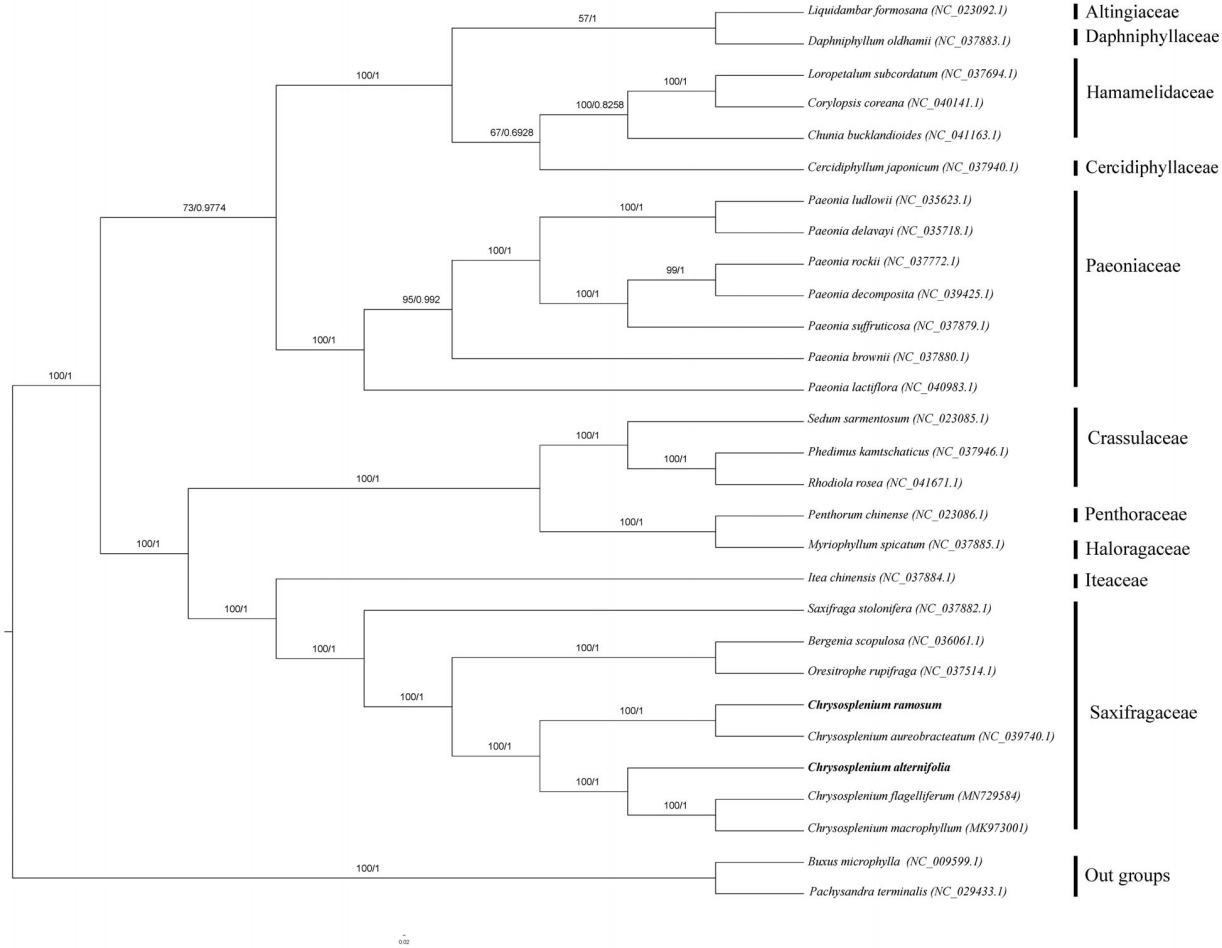
Phylogenetic tree reconstructed by maximum-likelihood (ML) and Bayesian inference (BI) analysis based on the whole chloroplast protein-coding genes of these 29 species.

## Data Availability

The data that support the findings of this study are openly available in Genbank (https://www.ncbi.nlm.nih.gov/genbank/) according to their Genbank accession.
